# Hot Air Impingement Drying Enhanced Drying Characteristics and Quality Attributes of *Ophiopogonis Radix*

**DOI:** 10.3390/foods12071441

**Published:** 2023-03-29

**Authors:** Zhian Zheng, Shanyu Wang, Chujie Zhang, Min Wu, Dezhou Cui, Xiaosong Fu, Lei Gao, Aichao Li, Qing Wei, Ziliang Liu

**Affiliations:** College of Engineering, China Agricultural University, Beijing 100083, China

**Keywords:** *Ophiopogonis Radix*, hot air impingement drying, drying characteristics, bioactive compounds, artificial neural network

## Abstract

The effects of drying temperature and air velocity on the drying characteristics, color, bioactive compounds, rehydration ratio, and microstructure of *Ophiopogonis Radix* during hot air impingement drying (HAID) were explored in the current study. The experimental results showed that the drying temperature and air velocity had a significant impact on the drying characteristics and quality attributes of dried products except for the rehydration ratio. The drying time decreased from 720 to 240 min with the increase of drying temperature from 50 to 70 °C. Increasing the air velocity from 6 to 12 m/s enhanced the drying process of *Ophiopogonis Radix*, while the extension of air velocity to 15 m/s lowered the drying rate. The samples that were dried at a lower drying temperature obtained lower color difference. Properly increasing the drying temperature or air velocity could increase the total polysaccharide and flavonoid contents of dried products. Additionally, a back-propagation neural network (BPNN) model was developed to predict the moisture ratio of *Ophiopogonis Radix* during the drying process. The optimal BPNN with 3-11-1 topology were obtained to predict the moisture ratio of *Ophiopogonis Radix* during HAID and performed with an acceptable performance.

## 1. Introduction

*Ophiopogonis Radix* is the tuberous root of *Ophiopogon japonicus* (Linn. f.) Ker Gawl., which is mainly cultivated in Southeast Asia, especially in most areas of China including Sichuan, Zhejiang, and Hubei provinces. The dried rhizome is a well-known traditional Chinese medicinal material with a history of 2000 years. *Ophiopogonis Radix* is renowned for its various bioactive components such as polysaccharides, homoisoflavonoids, saponins, amides, and monoterpene glycosides, which contribute to nourishing yin, moistening the lungs, treating inflammatory diseases, treating diabetes, and preventing aging [1,2,3]. Due to the seasonal properties and high moisture content of the fresh *Ophiopogonis Radix*, further processing after harvesting must be conducted to extend its availability out of season.

Drying is the most frequently used method to extend the shelf life of *Ophiopogonis Radix* by reducing the moisture content to a certain level. It can also reduce the weight and volume of materials and thus minimize the cost of transportation and storage [4]. Open sun drying is still practiced for *Ophiopogonis Radix* processing, which requires high labor costs and leads to contamination and uneven shrinkage of products. Moreover, the open sun drying method usually results in long drying time, rotting caused by the poor weather, and insect pollution [5]. Hot air convective drying (HAD) is the method that is mainly used in industrialized production of agro-materials due to its low operating cost. However, HAD of *Ophiopogonis Radix* is time consuming with low energy efficiency, especially in the falling-rate period. The prolonged exposure to elevated air temperature might lead to substantial deterioration of samples’ quality including structural changes, severe shrinkage, and the loss of bioactive compounds and color [6]. To improve the drying process of *Ophiopogonis Radix*, several researchers have applied different alternatives, such as microwave-, vacuum-, and freeze-drying for *Ophiopogonis Radix* preservation [7,8]. Although microwave drying performed high drying efficiency with low energy consumption, it may cause surface overheating due to the uneven heat distribution [9]. Vacuum- and freeze-drying can greatly reduce the loss of bioactive components and color of dried products [10], whereas the drying process that is required is a relatively long time with more energy with a lower production efficiency.

Among various drying methods, hot air impingement drying (HAID) can be considered as a good alternative to enhance the drying process of *Ophiopogonis Radix* with the improvement of product quality attributes. During the HAID process, the hot air directly impinges on the surface of materials through annular nozzles with high velocity, which can remove the thermal boundary layers over the material surface and thus promote the heat transfer [11]. It has been reported that the coefficient of heat transfer during HAID is about five times higher than that of cross-circulation drying, which greatly increases the drying efficiency [12]. With these advantages, HAID was employed to process American ginseng slices and the results showed that the proper drying temperature can extensively retain color and bioactive ginsenosides of dried product with a high drying rate [13]. Ai et al. [14] compared the performances of HAD and HAID in processing *Amomum villosum* fruits. They found that the hot air impingement on the dried products obtained higher total flavonoid content and DPPH values with a lower drying time and energy consumption [14]. Moreover, air impingement drying technology has been successfully applied in processing red pepper [15], apricot [16], broccoli florets [17], orange peel [18], and potato cubes [19]. However, to the best of our knowledge, there is limited information about the effect of HAID on the drying characteristics and quality attributes of *Ophiopogonis Radix* during drying.

The moisture content of the materials is an important index to determine the drying end point during the drying process. Currently, many mathematical models, including theoretical, empirical, and semi-empirical models were applied to predict the drying process [20]. However, the drying process was usually highly non-linear and dynamic and those mathematical models could only get accurate results under specific drying conditions. An artificial neural network (ANN) model can be a good alternative method to predict moisture content. ANN models showed better robustness and generalization performance than other mathematical models in fitting the drying process [21,22]. Various researchers have developed ANN-based models to predict the change of moisture content of different agro-materials during the drying process including grapes [21], potatoes [19], kiwifruit slices [23], and broccoli florets [17].

Drying parameters directly influence the drying efficiency and quality of the dried product. The selection of drying temperature and air velocity during HAID should be based on the rate of water migration and stability of the chemical composition to heat. On the other hand, the rate of water evaporation and microstructural changes of materials during drying may directly impact the rehydration characteristics of the dried products [24]. Therefore, the aims of the current work are to (1) determine the effects of drying temperature and air velocity on the drying characteristics of *Ophiopogonis Radix* under HAID; (2) evaluate the changes in the quality attributes of *Ophiopogonis Radix* in terms of color, total polysaccharide content, total flavonoid content, microstructure, and rehydration ratio; and (3) establish an ANN model to predict the moisture content of *Ophiopogonis Radix* during HAID. This study is expected to provide valuable information to promote the development of feasible practical solutions for processing *Ophiopogonis Radix*.

## 2. Materials and Methods

### 2.1. Materials

Fresh *Ophiopogonis Radix* samples were harvested from Santai planting base of Mianyang, Sichuan Province, China. The same batch of defect-free samples with uniform size were selected as a test material. The fresh materials were stored in the refrigerator at a temperature of 4 °C and a relative humidity of 90% in a refrigerator. The samples were taken from the refrigerator and brought to room temperature (25 °C) (China Agricultural University, Beijing, China) for two hours before the experiments. The initial moisture content of *Ophiopogonis Radix* on a wet basis (w.b.) was 61.86 ± 0.76%, which was determined by vacuum drying at 70 °C for 24 h following the AOAC standard method [25]. The dried *Ophiopogonis Radix* samples were ground into powder and passed through a 60-mesh sieve. The obtained powders were stored at −20 °C for analyses of quality attributes.

### 2.2. Experimental Procedure

**Figure 1:**
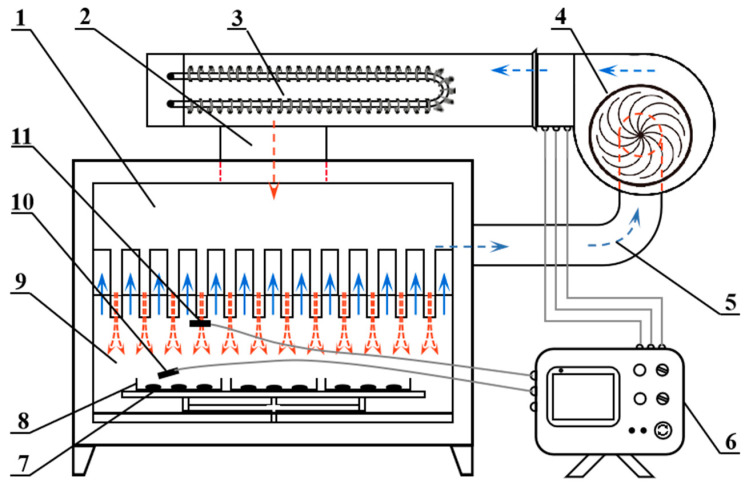
Schematic diagram of the hot air impingement dryer. 1. Airflow distribution chamber; 2. drying air channel; 3. electric heater; 4. centrifugal fan; 5. drying air recycle channel; 6. temperature sensor of the controller; 7. sample; 8. sample tray; 9. drying chamber with series of round nozzles; 10. temperature sensor; and 11. air velocity sensor.

The hot air impingement dryer (HAID) installed in the College of Engineering of China Agricultural University, Beijing, China, was used to conduct the *Ophiopogonis Radix* drying experiments. A schematic diagram of the dryer is displayed in Figure 1. It mainly contains an electric heater, proportional-integral-derivative (PID) controller, centrifugal fan, and a series of round nozzles. The distance between the round nozzles and the surface of the materials was approximately 12 cm. Due to the proximity of the nozzles to the drying product, the air velocity data that were collected by the hand-held airspeed sensor (TESTO-425, Testo Instruments International Trading (Shanghai) Co., Ltd., Shanghai, China) were measured to be in general agreement with the air velocity at the nozzle. After the air temperature in the drying chamber reached the target value at a steady state, about 100 g of prepared *Ophiopogonis Radix* samples were evenly spread on the stainless-steel wire grid in a single layer. The weight loss of the materials during drying was determined by moving out the tray and weighing on an electronic balance (SP402, Ohaus Co., Ltd., Pine Brook, NJ, USA) with an accuracy of ±0.01 g. The weighing process took less than 10 s. The drying process was continued until the moisture content of the samples was below 18% on a wet basis [26]. In the current work, the effects of two independent factors including drying temperature (50, 60, and 70 °C) and air velocities (6, 9, 12, 15 m/s) on drying characteristics and physicochemical properties of *Ophiopogonis Radix* were explored. All the drying experiments were conducted in triplicate.

### 2.3. Drying Characteristics

#### 2.3.1. Moisture Ratio

The moisture ratio (*MR*) of *Ophiopogonis Radix* at different times during drying was calculated by Equation (1) [27]:

(1) $$MR=\frac{M_{t}-M_{e}}{M_{0}-M_{e}}$$

where *M_e_*, *M*_0_, and *M_t_* indicate the equilibrium moisture content, initial moisture content, and moisture content at time *t*, respectively, g/g (dry basis, d.b.). Since the value of *M_e_* was relatively small compared to *M*_0_ and *M_t_*, Equation (1) could be simplified to Equation (2).

(2) $$MR=\frac{M_{t}}{M_{0}}$$

#### 2.3.2. Drying Rate

The drying rate (*DR*) reveals the moisture loss per unit time, which could be calculated using the following formula [27]:

(3) $$DR=\frac{M_{t1}-M_{t2}}{t_{2}-t_{1}}$$

where *t*_1_ and *t*_2_ indicate the drying time, min; and *M_t_*_1_ and *M_t_*_2_ denote the dry basis moisture content at time *t*_1_ and *t*_2_, respectively, g/g (d.b.).

#### 2.3.3. Effective Moisture Diffusivity

The effective moisture diffusivity (*D_eff_*) of *Ophiopogonis Radix* during drying can be calculated by one dimensional Fick’s second law with constant moisture diffusivity and negligible shrinkage phenomenon as shown in Equation (4) [19]:

(4) $$MR=\frac{M_{t}}{M_{0}}\approx \frac{8}{\pi^{2}}\exp\left(-\frac{\pi^{2}D_{eff}}{L^{2}}t\right)$$

where *D_eff_* indicates the effective moisture diffusivity, m^2^/s; *L* represents the thickness of samples, with 0.0050 m as its value; and *t* is the drying time for drying process, min.

Equation (4) could be simplified into Equation (5) with logarithmic form. The slope *k* of the curve can be calculated by linear regression method, and thus *D_eff_* can be obtained by substituting the slope *k* into Equation (6):

(5) $$\ln MR=\ln\frac{8}{\pi^{2}}-\frac{\pi^{2}D_{eff}}{L^{2}}t$$

(6) $$k=\frac{\pi^{2}D_{eff}}{L^{2}}$$

#### 2.3.4. Activation Energy

The drying activation energy (*E_a_*) refers to the connection between *D_eff_* and drying temperature can be generally described by the Arrhenius equation as shown in Equation (7) [18]:

(7) $$D_{eff}=D_{0}\exp\left[-\frac{E_{a}}{R(T+273.15)}\right]$$

where *D*_0_ is the effective moisture diffusivity basis, m^2^/s; *E_a_* is the drying activation energy, kJ/mol; *R* is the gas constant with a value of 8.314 J/(mol·K); and *T* means the drying temperature, °C.

Equation (7) can be represented by Equation (8) by taking the logarithm on both sides of the above formula. Therefore, *E_a_* can be obtained by the linear relationship between ln*D_eff_* and 1/(*T* + 273.15):

(8) $$\ln D_{eff}=\ln D_{0}-\frac{E_{a}}{R}\frac{1}{\left(T+273.15\right)}$$

### 2.4. Color Measurements

The surface color of the fresh and dried *Ophiopogonis Radix* was measured by a colorimeter (LabScan XE, Hunter Associates Laboratory, Inc., Reston, VA, America), which was quantified by CIE LAB color space coordinates, L* (lightness), a* (greenness/redness), and b* (blueness/yellowness) by CIE standard illuminant D65 and observer 10°. The range of L* is 0 (black)~100 (white), the range of a* is −60 (pure green)~60 (pure red), and the range of b* is −60 (pure blue)~60 (pure yellow). In addition, the color difference (Δ*E*) between the fresh and dried samples can be calculated by L*, a*, and b*, as shown in Equation (9) [16]:

(9) $$\Delta E=\sqrt{{\left(L^{*}-L_{0}^{*}\right)}^{2}+{\left(a^{*}-a_{0}^{*}\right)}^{2}+{\left(b^{*}-b_{0}^{*}\right)}^{2}}$$

where L*, a*, and b* are the measured values of the dried samples; and *L*_0_, *a*_0_, and *b*_0_ are the measured values of the fresh samples, respectively. All determinations were repeated at least six times.

### 2.5. Total Polysaccharides Content

The total polysaccharide contents of the samples were determined according to the method described by Xiao et al. [7] with slight modification. A total of 0.2 g powder sample of *Ophiopogonis Radix* was added into 50 mL of 80% aqueous ethanol and extracted at 80 °C for 1 h, then 50 mL of distilled water was added to extract at 100 °C for 2 h. The residues were extracted twice more. The supernatants were collected to 100 mL and diluted 25 times as sample solutions for later use. The absorbance of the extract was measured at 625 nm using a spectrophotometer (TU-1810, Beijing Purkinje General Instrument Co., Ltd., Beijing, China). 

### 2.6. Total Flavonoids Content

The total flavonoid contents in *Ophiopogonis Radix* were determined according to the method reported by Hossain and Gottschalk [28] with some modification. The powder (0.02 g) was added in the beaker with 2 mL of 60% aqueous ethanol and mixed well. The mixture was shaken for 2 h in a shaker (VORTEX-5, Changzhou Xiangtian Experimental Instrument Factory, Changzhou, China) with a speed of 270 rpm at a temperature of 60 °C. After that, the extracts were centrifuged at 10,000 rpm for 10 min by a centrifuge (GL-20G-II, Shanghai Anting Scientific Instrument Factory, Shanghai, China). The supernatants were collected for later use. The absorbance was measured at 502 nm using a spectrophotometer (TU-1810, Beijing Purkinje General Instrument Co., Ltd., Beijing, China).

### 2.7. Rehydration Ratio

The rehydration ratio (RR) was used to assess the water absorption ability of the dried *Ophiopogonis Radix* samples. The higher the RR value, the better the rehydration ability of the dried products. The rehydration potential was determined by soaking the samples in a water bath at 80 °C. Approximately 3.5 g of dried products were immersed into the 100 mL distilled water. The rehydration process was continued until the weight of the samples reached a constant value. During the rehydration process, the samples were taken out every 30 min and then the surface water was wiped off for weighing. The calculation formula of RR was shown as follows [29]:

(10) $$\mathrm{RR}=\frac{m_{e}}{m_{0}}$$

where RR is rehydration ratio, and *m_e_* and *m*_0_ are the weight of materials after and before rehydration, respectively, g.

### 2.8. Microstructure

The surface microstructure of dried *Ophiopogonis Radix* was determined using scanning electron microscope (JEOL, SU3500, Hitachi, Tokyo, Japan). The cuticular layer of the dried samples were cut into 3 × 3 mm pieces by a sharp razor blade and mounted on an aluminum base. The small pieces were coated with gold for about 50 s. The surface microstructure images were observed at the accelerating voltage of 15 kV. 

### 2.9. ANN Modeling

In current work, a supervised back-propagation neural network (BPNN) model was developed to predict the moisture ratio of *Ophiopogonis Radix* during HAID. The topological structure of BPNN consisted of three inputs (drying time, drying temperature, and air velocity), one hidden layer, and one output (moisture ratio). The sigmoid transfer function was selected to connect the hidden input and output layer. The learning rate, maximum number of training epochs, and sum of squared error were set to 0.01, 1000, and 0.0001, respectively. Since there was no general rule to select the number of hidden neurons, the stepwise searching method was applied to obtain the appropriate number of neurons in the hidden layer. A total of 70% of the datasets were applied for training and the other 30% were used for testing the BPNN model. The range of the number of hidden neurons in the training process was set in the range of 4 to 13 based on the number of inputs and outputs. The coefficient of determination (*R*^2^) and root mean square error (*RMSE*) values were determined by Equations (11) and (12) to check the performance of the models.

(11) $$R^{2}=1-\frac{\sum_{i=1}^{N}{\left(MR_{\mathrm{pre},i}-MR_{\exp,i}\right)}^{2}}{\sum_{i=1}^{N}{\left(\bar{MR_{\mathrm{pre},i}}-MR_{\exp,i}\right)}^{2}}$$

(12) $$RMSE={\left[\frac{1}{N}\sum_{i=1}^{N}{\left(MR_{\mathrm{pre},i}-MR_{\exp,i}\right)}^{2}\right]}^{\frac{1}{2}}$$

where *MR*_pre,*i*_ and *MR*_exp,*i*_ are experimental and predicted values; *N* means the number of data that were measured in the experiment; and *n* is the number of factor levels.

### 2.10. Data Analysis

The data analysis was conducted using Microsoft Excel 2016 (Microsoft Corporation, Redmond, WA, USA), SPSS statistics software (version 27.0, IBM Corp., Armonk, NY, USA) and Origin 2019 software (OriginLab Corp., Northampton, MA, USA). Statistically significant differences among the results were analyzed by one-way analysis of variance (ANOVA) and evaluated by the Tukey test with a significance level of 0.05. The experimental results were expressed as means ± standard deviation (SD).

## 3. Results and Discussion

### 3.1. Drying Kinetics

**Figure 2:**
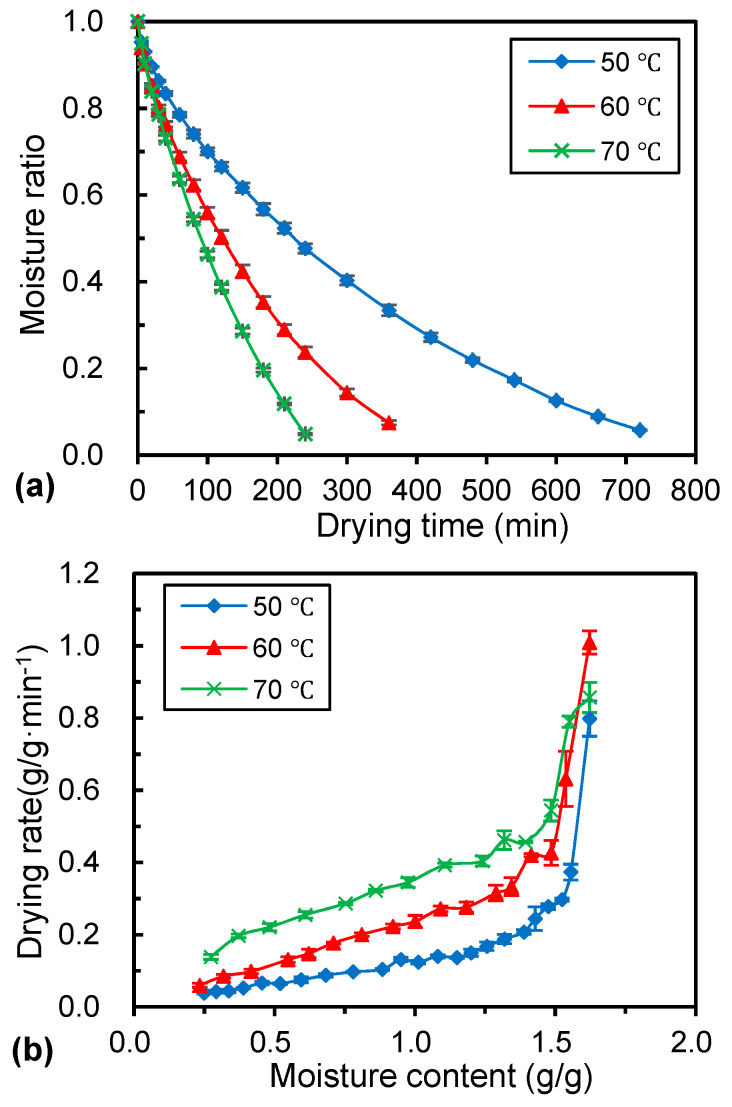
Drying kinetics (**a**) and drying rate (**b**) curves *Ophiopogonis Radix* under different drying temperatures.

The effect of drying temperature on the drying characteristics of *Ophiopogonis Radix* during HAID is plotted in Figure 2. As shown in Figure 2a, the moisture content of *Ophiopogonis Radix* gradually decreased with the extension of drying time. At the same air velocity of 12 m/s, the drying time significantly decreased with the increase of drying temperature and the drying time required for the desired moisture content was 720, 360, and 240 min for materials dried at temperatures of 50, 60, and 70 °C, respectively. The entire drying process also occurred in the falling-rate period (Figure 2b), which demonstrated that the internal moisture diffusion phenomenon impacted the drying process. The drying rate under different drying temperatures increased rapidly at the initial drying stage. This might be attributed to the presence of excessive moisture over the surface of the samples and the water removal rate was sufficiently high. With the removal of surface water, the drying rate gradually decreased. Additionally, the drying rate greatly increased with the increase of drying temperature. This is in agreement with a general perception that the larger heat transfer driving force can be generated at a higher drying temperature [6]. Similar results were obtained by Liu et al. [17] and Xiao et al. [13], who applied HAID in broccoli florets and American Ginseng slices processing, respectively.

### 3.2. Drying Rate

**Figure 3:**
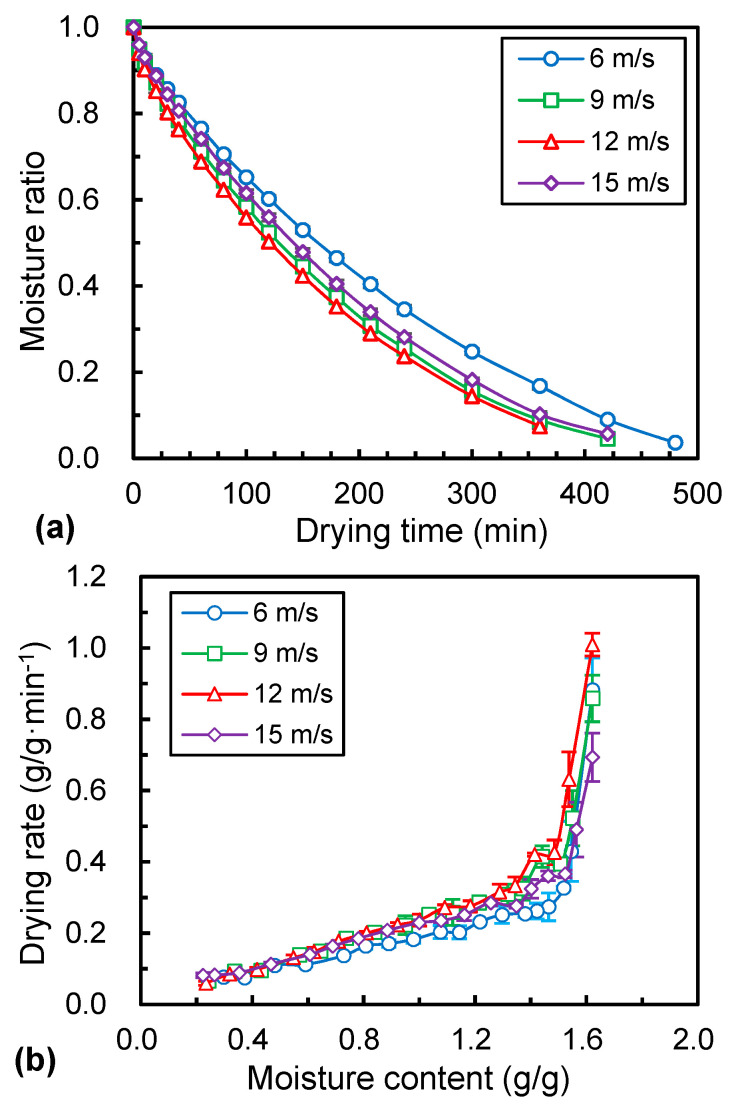
Drying kinetics (**a**) and drying rate (**b**) curves *Ophiopogonis Radix* under different air velocity.

Figure 3a shows the effect of air velocity on the drying curves of *Ophiopogonis Radix* at constant drying temperature of 60 °C. The drying time required for different air velocities, i.e., 6, 9, 12, and 15 m/s were 480, 420, 360, and 420 min, respectively. This means that the drying time decreased with the increase of air velocity from 6 to 12 m/s, while the drying time increased with the increase in air velocity from 12 to 15 m/s. This trend of drying rate under different air velocities was also similar with the drying time (Figure 3b). At the initial drying stage, the drying rate was limited by the water removal rate as the moisture content of the materials was relatively high. Therefore, increasing the air velocity to a certain extent could provide a larger volume of air over the surface of the samples, thus promoting the evaporation of water. However, the higher air velocity (15 m/s) could greatly enhance the evaporation of moisture over the materials’ surface and resulted in case hardening, so as to inhibit the moisture evaporation [30].

### 3.3. Effective Moisture Diffusivity and Activation Energy

The effective moisture diffusivity, *D_eff_*, of *Ophiopogonis Radix* during HAID under different drying conditions varied from 1.62 × 10^−10^ to 4.58 × 10^−10^ m^2^/s (Table 1), which is in the range of 10^−11^ to 10^−9^ m^2^/s for most agricultural products [31]. The *D_eff_* values increased with the increase of drying temperature. The change trends of *D_eff_* values under different air velocities were consistent with that of the drying time. The highest *D_eff_* of *Ophiopogonis Radix* was noted for the drying temperature of 70 °C with an air velocity of 12 m/s, while the lowest one was observed at the drying temperature of 50 °C with an air velocity of 12 m/s. The higher temperature accelerated the diffusion of water molecules and thus led to the higher effective moisture diffusivity.

The activation energy (*E_a_*) was considered as the energy that utilize to initiate moisture diffusion. *E_a_* value of *Ophiopogonis Radix* in current work was calculated to be 48.11 kJ/mol, which means that 48.11 kJ of energy was needed to evaporate one mole of water. It is also in the range of 12.7~110.0 kJ/mol for the most agro-materials.

### 3.4. Color Determination

Color is an important appearance index of dried products as it reflects the internal quality of products to a certain extent and affects consumers’ acceptability and preference [32]. The surface color parameters of fresh and dried materials under different drying conditions are given in Table 2. As shown in Table 2, drying treatment could significantly impact the L*, a*, and b* values (*p* < 0.05). Raising the drying temperature decreased the L* value and increased a* and b* values. This may indicate that the higher drying temperature might promote the interaction between reducing sugars and amino acids due to plentiful amino acids in *Ophiopogonis Radix* [33]. A higher temperature can also facilitate the Maillard reaction. As a result, the lowest Δ*E* value occurred in the dried samples at a drying temperature of 50 °C with an air velocity of 9 m/s. Moreover, appropriately increasing the air velocity could improve the color quality of the dried products. The air velocity of 6 m/s resulted in a long-time non-enzymatic reaction and lowered the lightness due to the extended drying time. The samples that were dried at a drying temperature of 65 °C with an air velocity of 6 m/s obtained the highest a*, b*, and Δ*E* values. These were in agreement with Liu et al. [10] and Wang et al. [34], who reported that the higher drying temperature or longer drying time was harmful to the color quality of the dried products.

### 3.5. Total Polysaccharides Content

**Figure 4:**
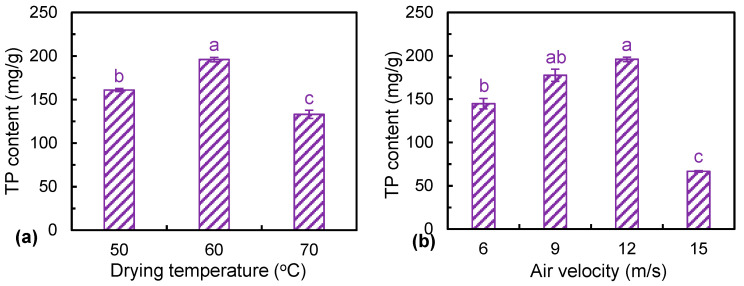
Total polysaccharide content of dried *Ophiopogonis Radix* under different drying temperatures (**a**) and air velocities (**b**). Different letters (a–c) indicate the significant differences at *p* < 0.05.

Figure 4 presents the values of the total polysaccharide content of dried *Ophiopogonis Radix* under different drying conditions. Drying treatment could greatly reduce the total polysaccharide content of *Ophiopogonis Radix*. The total polysaccharide content of HAID samples was in the range of 66.70 to 196.03 mg/g. Both the drying temperature and air velocity had a significant impact on the total polysaccharides content of *Ophiopogonis Radix* (*p* < 0.05). From Figure 4a, the highest total polysaccharide content, i.e., 196.03 mg/g, was noted at 60 °C, while the increase of the drying temperature from 60 to 70 °C obviously decreased the total polysaccharide content. This might be due to the fact that polysaccharides are sensitive to heat and the higher drying temperature resulted in thermal degradation of the polysaccharides [35]. This finding was consistent with the results that were reported by Xie et al. [36] in processing wolfberry. The appropriate increase of air velocity could also significantly enhance the retention of polysaccharides (Figure 4b). The total polysaccharide content of dried products at different air velocities of 6, 9, 12, and 15 m/s were 144.73, 177.63, 196.03, and 66.70 mg/g, respectively. The increase of the air velocity from 6 to 12 m/s could greatly reduce the drying time and thus shorten the exposure time of samples to the heat.

### 3.6. Total Flavonoids Content

**Figure 5:**
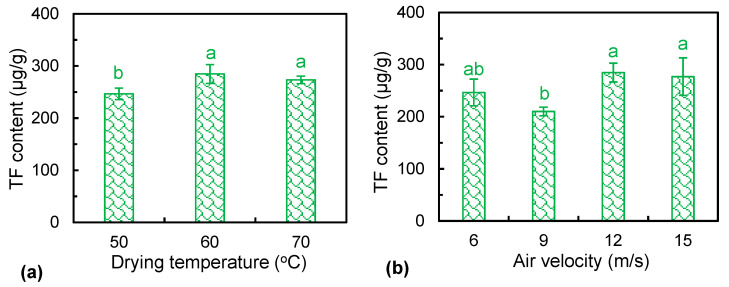
Total flavonoid content of dried *Ophiopogonis Radix* under different drying temperatures (**a**) and air velocities (**b**). Different letters (a–b) indicate the significant differences at *p* < 0.05.

*Ophiopogonis Radix* is rich in flavonoids, which play an essential role in scavenging reactive oxygen species [37]. Figure 5 depicts the total flavonoid content of dried *Ophiopogonis Radix* under different drying temperatures and air velocities. As shown in Figure 5a, the content of flavonoids increased at first and then decreased with the increase in temperature. The lowest total flavonoid content, i.e., 210.12 μg/g, occurred in the samples dried at the temperature of 50 °C. This might be due to the fact that a lower drying temperature prolonged the exposure time to the heat and promoted the thermal degradation of flavonoid components. Although the flavonoids were sensitive to heat, high temperatures could greatly reduce the drying time and thus reduce enzymatic oxidation and thermal degradation. It is noted that there was no significant influence on the total flavonoid content between 60 and 70 °C. In addition, increasing the air velocity could increase the total flavonoid content of dried materials (Figure 5b). The air velocity of 12 m/s produced the dried products with the highest total flavonoid content, i.e., 284.71 μg/g. Increasing the air velocity to some extent could shorten the drying time and thus reduce the thermal degradation of flavonoid components [30].

### 3.7. Rehydration Ratio

**Figure 6:**
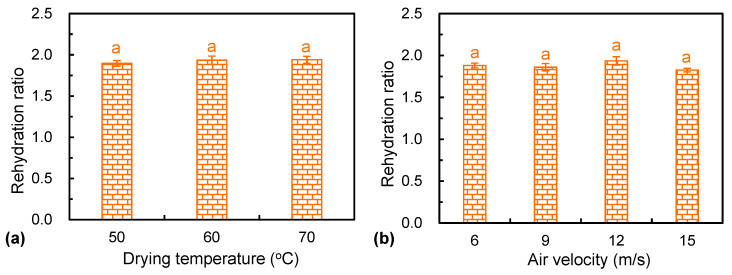
Rehydration ratio of dried *Ophiopogonis Radix* under different drying temperatures (**a**) and air velocities (**b**). The letter (a) indicates that there is no significant difference at *p* < 0.05.

**Figure 7:**
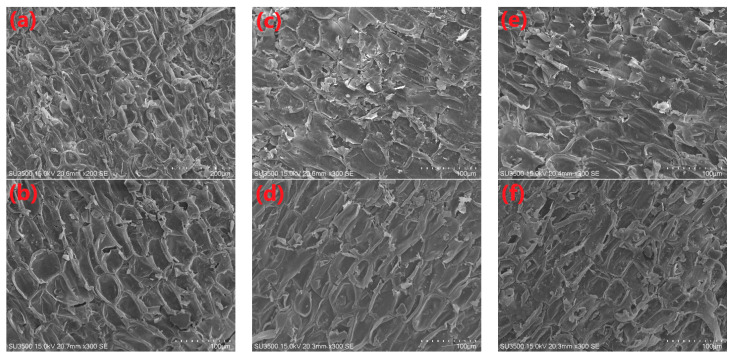
SEM micrographs of dried *Ophiopogonis Radix*. (**a**) Dried at 50 °C with 12 m/s, (**b**) dried at 60 °C with 12 m/s, (**c**) dried at 70 °C with 12 m/s, (**d**) dried at 60 °C with 6 m/s, (**e**) dried at 60 °C with 9 m/s, and (**f**) dried at 60 °C with 15 m/s.

The rehydration performance is a major index to evaluate the extent of damage on the cell structure of dried materials [17]. The experimental results of the rehydration under various drying parameters are shown in Figure 6. No significant differences (*p* > 0.05) were found in rehydration capacities among the samples that were dried at different drying temperatures and air velocities. This phenomenon might be due to the fact that the sugars inside the materials moved to the surface under heat stress. Different drying conditions might cause the same level of damage on the structure of *Ophiopogonis Radix*. As can be seen from Figure 7, the cell skeleton structure at the cuticular layer was damaged to a certain extent under different drying conditions. There was no significant difference in the microstructure between different conditions. A similar phenomenon was found by Liu et al. [26], who applied pulsed vacuum drying technology for processing kiwifruit slices. Contrary to this, the HAID conditions could significantly impact the rehydration properties of American ginseng slices [13]. The different results of the rehydration ratio under the same drying technology might be due to the differences in the materials’ structure.

### 3.8. ANN Modeling Results

The BPNN was developed to predict the moisture ratio of *Ophiopogonis Radix* during HAID. In order to avoid the over fitting problem, all of the obtained datasets were normalized in the range of 0 to 1 according to Equation (13):

(13) $$y_{n}=\frac{y_{i}-y_{\min}}{y_{\max}-y_{\min}}$$

where *y*_max_, *y*_min_, *y_n_*, and *y_i_* indicate the maximum, minimum, normalized, and experimental values of features, respectively.

**Figure 8:**
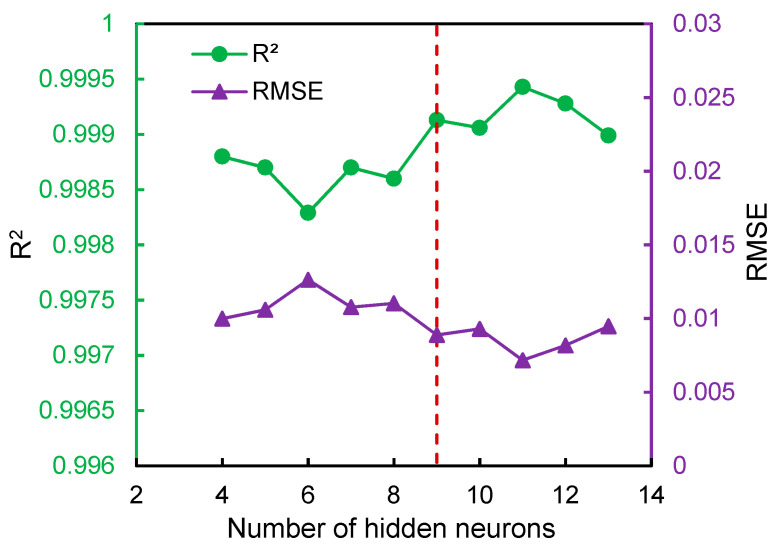
The training performance of the BPNN model under different numbers of hidden neurons.

**Figure 9:**
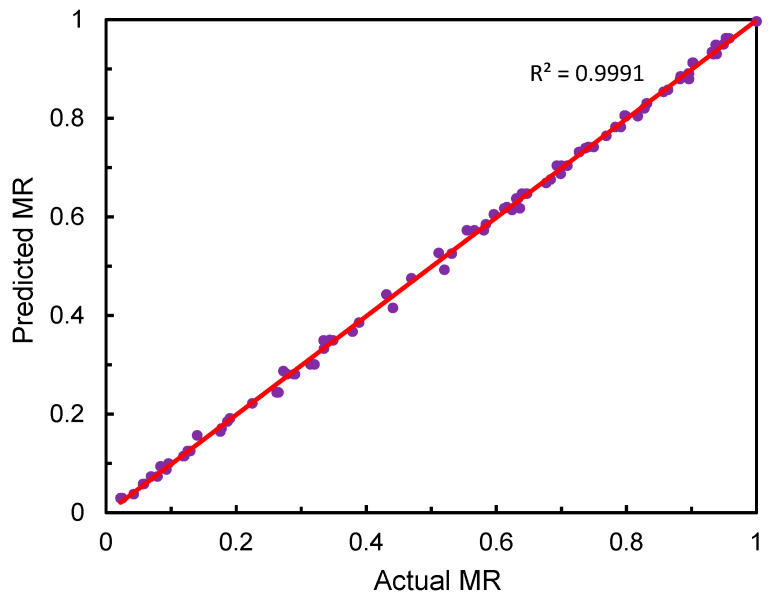
The predicted vs. actual MR for testing datasets.

Figure 8 illustrates the training performance of BPNN in terms of *R*^2^ and *RMSE* under a different number of hidden neurons. Generally, the lower value of RMSE and higher value of *R*^2^ of the BPNN model indicate the better training performance. From Figure 8, it can be obviously seen that the highest *R*^2^ and lowest *RMSE* occurred when there were 11 hidden neurons. The corresponding values of *R*^2^ and *RMSE* were 0.9994 and 0.0072, respectively. In terms of *R*^2^ value, the performance of BPNN was close to the measured value in the reported case [38]. Therefore, the optimal BPNN with 3-11-1 topology were selected to predict the moisture ratio of *Ophiopogonis Radix* during HAID. The fitness between the actual moisture ratio and the predicted values are shown in Figure 9. The actual and predicted values almost coincided and the *R*^2^ and *RMSE* were 0.9991 and 0.0092, respectively, which indicated that the developed BPNN had a high level of accuracy in predicting the moisture ratio of *Ophiopogonis Radix* during HAID.

## 4. Conclusions

The experimental results indicated that the drying time decreased from 720 to 240 min with the increase of drying temperature from 50 to 70 °C. The drying rate increased with the increase of air velocity from 6 to 12 m/s, while the extension of the air velocity to 15 m/s lowered down the drying rate of *Ophiopogonis Radix*. The lower the drying temperature, the lower the color difference between the dried products. Properly increasing the drying temperature or air velocity could increase the total polysaccharide and flavonoid contents of dried *Ophiopogonis Radix*. Both the highest total polysaccharide and flavonoid contents of dried products, i.e., 196.03 mg/g and 284.71 μg/g, were obtained at the drying temperature of 60 °C with an air velocity of 12 m/s. However, there was no significant influence on the rehydration ratio of dried samples between different drying conditions. Additionally, the optimal BPNN with 3-11-1 topology was determined to predict the moisture ratio of *Ophiopogonis Radix* during HAID. *R*^2^ and *RMSE* between the predicted and actual values were 0.9991 and 0.0092, respectively. Overall, the results revealed that HAID can be a promising technology for processing *Ophiopogonis Radix* with improved drying efficiency and quality of products. In the future, the effects of different loading capacity of materials on the drying characteristics and multi-objective optimization of drying processes will be examined, so as to provide more information for the industrialized processing of *Ophiopogonis Radix*.

## Figures and Tables

**Table 1:** Effective moisture diffusion coefficients and activation energy.

Parameter	Condition	Linear Regression Equation	*R* ^2^	*D_eff_* (10^−10^ m^2^/s)	*E_a_* (kJ/Mol)
Temperature (°C)	50	ln*MR* = −6.4 × 10^−5^*t* + 0.0695	0.9600	1.62	48.11
	60	ln*MR* = −1.36 × 10^−4^*t* + 0.1572	0.9165	3.44	
	70	ln*MR* = −1.81 × 10^−4^*t* + 0.1303	0.9463	4.58	
Air velocity (m/s)	6	ln*MR* = −9.8 × 10^−3^*t* + 0.1172	0.9489	2.48	
	9	ln*MR* = −1.24 × 10^−4^*t* + 0.1347	0.9377	3.14	
	12	ln*MR* = −1.36 × 10^−4^*t* + 0.1572	0.9165	3.44	
	15	ln*MR* = −1.12 × 10^−4^*t* + 0.1174	0.9555	2.84	

**Table 2:** Color parameters of raw and dried *Ophiopogonis Radix* samples.

Parameter	Condition	L*	a*	b*	Δ*E*
Fresh	-	87.60 ± 0.08 ^a^	−0.46 ± 0.03 ^d^	7.60 ± 0.03 ^e^	-
Temperature (°C)	50	84.77 ± 0.08 ^b^	0.44 ± 0.03 ^c^	8.71 ± 0.10 ^d^	3.17 ± 0.10 ^f^
	60	80.37 ± 0.16 ^e^	0.89 ± 0.03 ^a^	12.00 ± 0.21 ^b^	8.57 ± 0.24 ^c^
	70	78.28 ± 0.04 ^f^	0.71 ± 0.09 ^b^	14.13 ± 0.18 ^a^	11.44 ± 0.14 ^b^
Air velocity (m/s)	6	77.12 ± 0.31 ^g^	1.00 ± 0.13 ^a^	14.44 ± 0.29 ^a^	12.59 ± 0.43 ^a^
	9	83.24 ± 0.18 ^c^	0.47 ± 0.04 ^c^	10.45 ± 0.23 ^c^	5.29 ± 0.28 ^e^
	12	80.37 ± 0.16 ^e^	0.89 ± 0.03 ^a^	12.00 ± 0.21 ^b^	8.57 ± 0.24 ^c^
	15	82.38 ± 0.10 ^d^	0.72 ± 0.04 ^b^	10.66 ± 0.12 ^c^	6.16 ± 0.15 ^d^

Note: Different letters (^a–g^) in the same column indicate the significant differences at *p* < 0.05.

## Data Availability

The data presented in this study are available on request from the corresponding author.
